# Deciphering the Matrisome: Extracellular Matrix Remodeling in Liver Cirrhosis and Hepatocellular Carcinoma

**DOI:** 10.7759/cureus.82171

**Published:** 2025-04-13

**Authors:** Ram Prasad Chaulagain, Aadil Mushtaq Padder, Harisharan Shrestha, Radheshyam Gupta, Rameshor Bhandari, Yelona Shrestha, Abdulkareem Qasem Moqbel, Smriti Gautam, Nand Lal, Shizhu Jin

**Affiliations:** 1 Internal Medicine, Second Affiliated Hospital of Harbin Medical University, Harbin, CHN; 2 Gastroenterology and Hepatology, Second Affiliated Hospital of Harbin Medical University, Harbin, CHN; 3 Emergency, Civil Service Hospital of Nepal, Kathmandu, NPL; 4 Urology Surgery, Cancer Hospital, Harbin Medical University, Harbin, CHN; 5 Surgical Gastroenterology, Grande International Hospital, Kathmandu, NPL; 6 Dermatology, First Affiliated Hospital of Xinjiang Medical University, Xinxiang, CHN; 7 College of Medical Science, Taiz University, Taiz, YEM; 8 Dermatology, Kathmandu Medical College, Kathmandu, NPL; 9 Physiology, School of Biomedical Sciences, Harbin Medical University, Harbin, CHN

**Keywords:** extracellular matrices, hepatocellular carcinome, liver cirrhosis, matrisome, matrix metalloproteinases

## Abstract

Liver cirrhosis and hepatocellular carcinoma (HCC) are major public health concerns due to their high morbidity and mortality rates. The liver, a vital organ for metabolism, detoxification, and homeostasis, depends on the matrisome, a complex and dynamic network of extracellular matrix (ECM) components for maintaining structural and functional integrity. Chronic liver inflammation, induced by factors such as alcohol abuse, viral hepatitis, and non-alcoholic fatty liver disease, leads to fibrosis and cirrhosis, progressing to HCC. The matrisome, composed of ECM proteins including collagen, fibronectin, and laminin, plays a critical role in regulating tissue homeostasis, cell signaling, and tissue repair. Dysregulation of ECM components contributes to the pathogenesis of both liver cirrhosis and cancer. In cirrhosis, matrisome alterations are characterized by excessive ECM deposition and fibrosis, which disrupt the liver’s architecture and impair its function. Activated hepatic stellate cells (HSCs) are the principal mediators of fibrosis, producing large quantities of ECM components. In liver cancer, matrisome remodeling facilitates tumorigenesis by promoting cancer cell proliferation, invasion, and metastasis. The tumor microenvironment, shaped by ECM alterations, further supports tumor growth and dissemination. Matrix metalloproteinases (MMPs) play a pivotal role in ECM degradation, fibrosis progression, and tumor invasion, while tissue inhibitors of metalloproteinases (TIMPs) modulate MMP activity. A comprehensive understanding of the molecular mechanisms that link matrisome alterations with the progression from cirrhosis to liver cancer is essential for identifying novel diagnostic and therapeutic targets. This review highlights the dynamic responses of the hepatic matrisome to both acute and chronic insults, emphasizing the complex interplay between ECM components, cellular behavior, and disease progression. Elucidating these interactions may inform strategies aimed at improving clinical outcomes for patients with liver cirrhosis and HCC.

## Introduction and background

**Figure 1:**
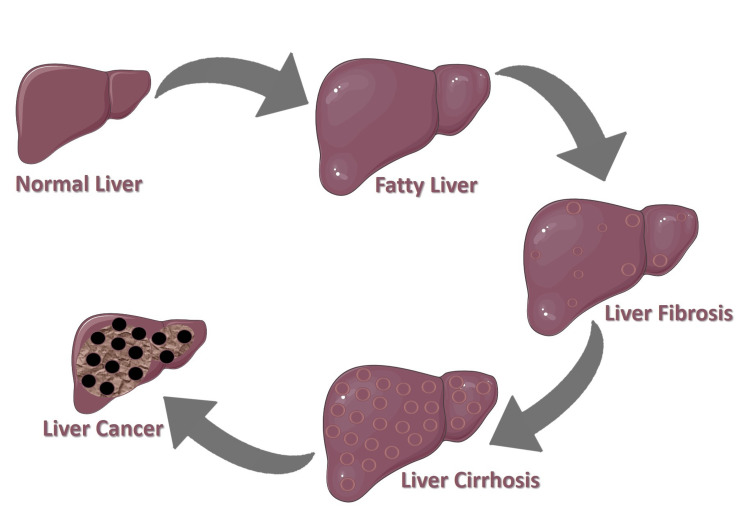
Progressive pathological changes in liver disease Credit: Adapted from Servier Medical Art [8], licensed under Creative Commons Attribution License (CC BY 4.0).

Liver cirrhosis is a major public health issue, associated with substantial morbidity and mortality. The liver, one of the most vital organs in the human body, performs essential functions related to metabolism, detoxification, and systemic homeostasis [1]. Matrisome, a complex and dynamic extracellular matrix (ECM) that constitutes the structural framework of liver tissue, plays a vital role in maintaining its structural and functional integrity. In addition to providing mechanical support, the matrisome also serves as a repository for signaling molecules, growth factors, and cytokines [2]. Liver cirrhosis is characterized by chronic hepatic inflammation and progressive liver tissue damage. Alcoholism, viral hepatitis, non-alcoholic fatty liver disease, autoimmune conditions, and metabolic abnormalities are primary causes of liver cirrhosis [3]. Advanced liver cancer is characterized by diffuse fibrosis, architectural distortion, and the replacement of functional hepatic parenchyma with nonfunctional scar tissue. This pathological process typically progresses from chronic liver disease to fibrosis and ultimately to cirrhosis, which significantly increases the risk of hepatocellular carcinoma (HCC) [4]. Liver fibrosis represents an aberrant wound healing response, characterized by the excessive accumulation of collagen and other ECM components [5]. When fibrosis becomes extensive and irreversible, it progresses to cirrhosis, a severe stage of chronic liver disease that is defined by the formation of regenerative nodules and distortion of the hepatic vasculature. These structural changes lead to impaired liver function, portal hypertension, and further increase the risk of HCC development [6]. The matrisome, a collective term for the ensemble of ECM proteins and associated factors, plays a pivotal role in maintaining tissue architecture and homeostasis and is critically involved in fibrogenic remodeling during chronic liver injury [7]. Liver cirrhosis and HCC are critical pathological processes significantly impacting liver health (Figure 1).

Tumorigenesis is the process by which cells develop abnormally and uncontrollably to form tumors. The matrisome facilitates cancer cell proliferation, invasion, and metastasis, thereby promoting tumorigenesis. Poor patient survival and the development of tumorigenesis and metastasis may be caused by matrisome proteins produced by cancer cells [9]. Matrisome genes undergo various alterations and mutations, which affect gene expression and protein function, leading to tumorigenesis [10]. Additionally, hypoxia, acidosis, oxygen free radicals produced by invading inflammatory cells, or proteases secreted by tumor or stromal cells can all induce modifications in the ECM, including the matrisome [11]. The tumor matrisome index (TMI) demonstrates how cancer progresses due to alteration of the matrisome [12]. Chronic liver injury can result in the accumulation of extracellular proteins, ultimately leading to the development of liver cirrhosis [13]. Interestingly, it is hypothesized that inhibiting low levels of fibronectin during hepatic fibrogenesis could reduce collagen accumulation and improve liver function, potentially halting the progression of liver cirrhosis [14].

The ECM comprises a diverse array of components that interact bidirectionally with the surrounding cells, creating a dynamic and valuable microenvironment that regulates cellular and tissue homeostasis [15]. The ECM comprises various fibrous proteins such as collagen, glycoproteins, and proteoglycans. Collagen-associated proteins, ECM-regulating and modifying enzymes (e.g., lysyl oxidases and proteases), and ECM-resident storage molecules such as transforming growth factor-beta (TGF-β) and other cytokines collectively constitute what is referred to as the matrisome [16]. The ECM functions as a reservoir for growth factors, cytokines, and signaling molecules, enabling bidirectional communication between cells and their microenvironment. It also provides essential structural support for various cell types, including stem cells. Dysregulation of ECM components has been implicated in a range of pathological conditions, including cancer, genetic disorders, and abnormal stem cell behavior [17]. Moreover, the ECM provides mechanical support to organs and tissues, maintaining their structural integrity [18]. ECM proteins comprise independently folded regions characterized by highly conserved sequences and structures. These domains interact with adhesion receptors such as integrins to transmit signals to cells and facilitate cell-matrix attachment [19]. This review explores the importance of understanding ECM-associated proteins and the matrisome in exploring biological diversity, with a particular focus on the mechanisms underlying liver cirrhosis and cancer.

Studies have found that the hepatic matrisome exhibits dynamic responses to acute lipopolysaccharide (LPS) exposure and chronic ethanol-induced stress well before the appearance of overt fibrotic changes in the liver. The pathological responses to these stressors may, in part, be driven by alterations in the matrisome [20]. It should be noted that cirrhosis is highly prevalent among patients with HCC, regardless of the underlying liver disease [21]. However, the precise molecular relationship between cirrhosis and HCC, particularly in the context of matrisome alterations, remains inadequately defined. Given the global burden of cirrhosis and liver cancer, this represents a critical area of clinical interest. Furthermore, the involvement of matrisome remodeling in tissue reorganization and tumorigenesis highlights a compelling direction for future research. A comprehensive understanding of these interactions may inform the development of novel diagnostic and therapeutic strategies, ultimately improving outcomes for individuals affected by these debilitating diseases.

## Review

Significance of the matrisome in liver health, homeostasis, and disease

Tissue Organization and ECM-Matrisome Dynamics

**Figure 2:**
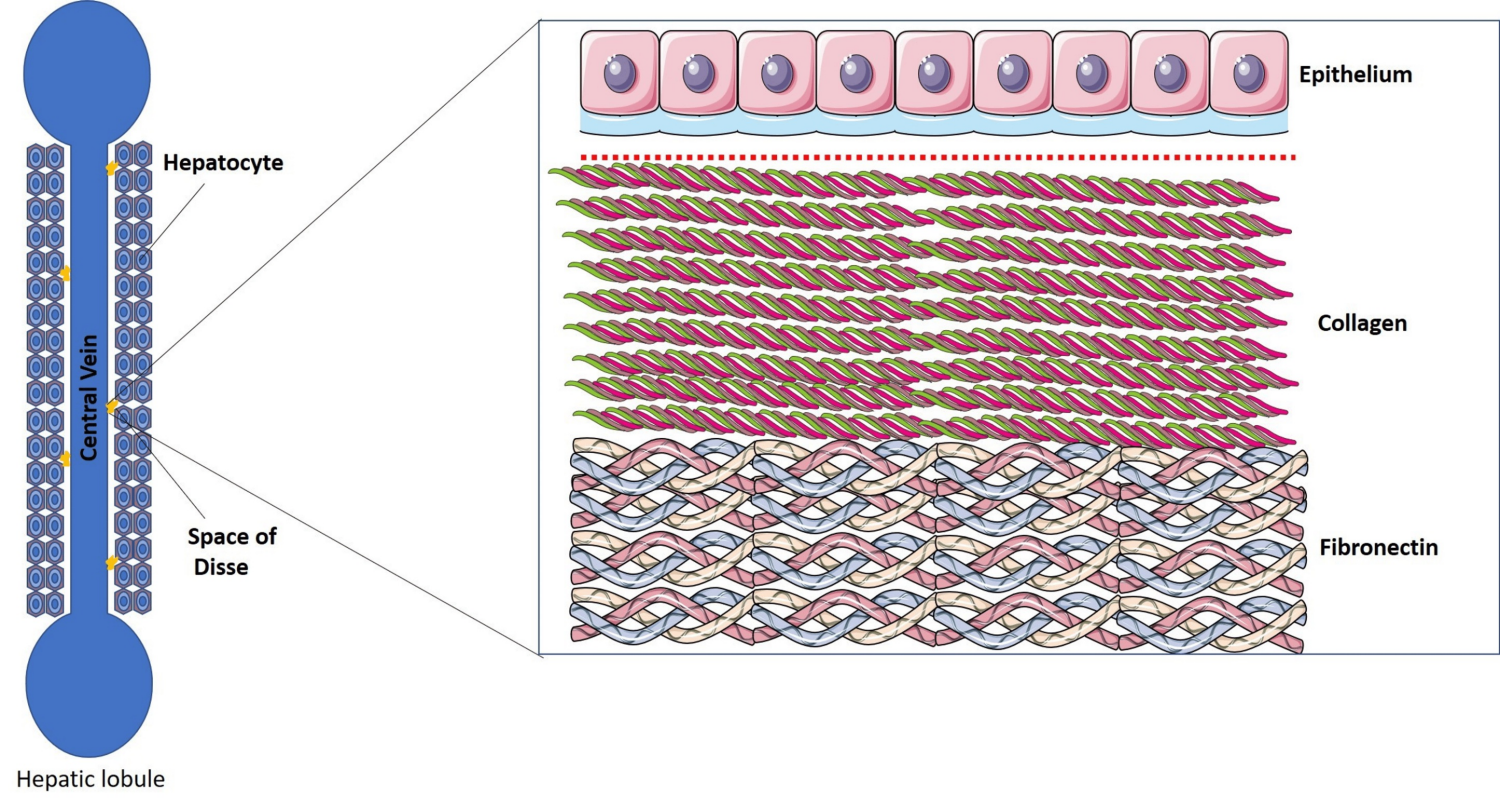
Structural organization of hepatic matrisome Credit: Adapted from Servier Medical Art [8], licensed under Creative Commons Attribution License (CC BY 4.0).

The ECM, composed of a network of proteins and polysaccharides, supports a wide range of biological structures and functions (Figure 2), including tissue development, mechanical flexibility, and the maintenance of structural integrity across entire organs [22]. The ECM, which contributes significantly to the volume, shape, and mechanical strength of many tissues in vivo [23]. In the liver, the ECM plays a central role in preserving its functional, physiological, and anatomical architecture [24]. It serves not only as a structural scaffold but also as an active regulator of cellular processes [25].

Maintaining Homeostasis

The matrisome provides tissue homeostasis by controlling cell behavior and preserving an ideal microenvironment. The ECM interacts with cells through cell adhesion receptors like integrins, influencing cellular processes including survival, proliferation, and differentiation. To maintain a balance between cell development and apoptosis, it offers biochemical and mechanical cues that guide cellular responses to external stimuli [26]. In the liver, it plays a pivotal role in regulating key physiological processes essential for homeostasis, such as blood flow, nutrient exchange, and waste elimination [27]. 

Cell Signaling

Different growth factors, cytokines, and signaling chemicals are stored in the matrisome. These bioactive compounds can be sequestered within the ECM and subsequently released in a regulated manner to modulate cellular behavior. Additionally, the physical properties of the ECM, such as stiffness and rigidity, influence intracellular signaling cascades. Gene expression, cell migration, and cytoskeletal rearrangements are impacted by the bidirectional signaling between the ECM and cells made possible by integrins and other ECM receptors [28].

Tissue Repair

The matrisome is essential for tissue regeneration and repair. The ECM offers a scaffold that directs cell migration and encourages tissue rebuilding following tissue injury. During wound healing and clot formation, the ECM incorporates bioactive components such as fibronectin and fibrin [29]. The matrisome goes through dynamic changes during tissue repair, with ECM remodeling involving the breakdown of old matrix components and the synthesis of new ones [30]. In the context of hepatic injury, the matrisome and ECM are indispensable for orchestrating effective tissue repair and regeneration [31].

Involvement in Liver Diseases

The role of the ECM in the early stages of chronic liver disease remains incompletely understood. However, recent studies have shown that various changes in the hepatic ECM in fibrotic liver disease are present and may also impact the course of the disease [32]. For instance, excessive ECM deposition in cirrhosis might result in liver scarring and reduced liver function.

Tumor Microenvironment

The ECM plays a significant role in the tumor microenvironment (TME) in the setting of liver cancer. Up to 60% of tumor mass can be made up of solid tumors, which can impact metastasis, invasion, and tumor growth [33].

Matrisome alteration in liver cirrhosis

**Figure 3:**
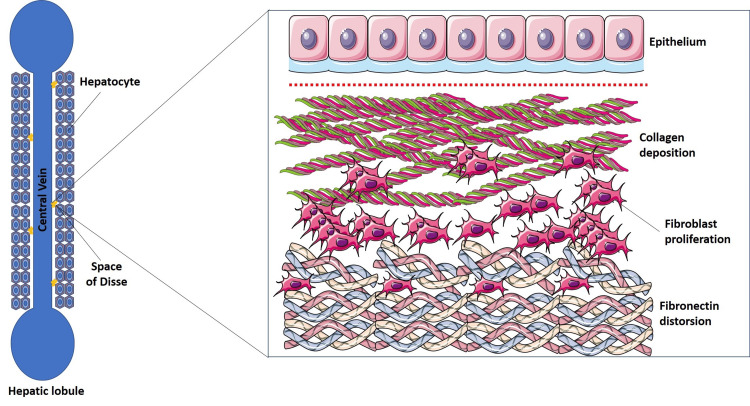
Matrisomal alteration in liver cirrhosis Credit: Adapted from Servier Medical Art [8], licensed under Creative Commons Attribution License (CC BY 4.0).

ECM proteins build up over time, the hallmark of the degenerative liver disease known as liver cirrhosis. Matrisome modifications are changes in the ECM within the liver tissue that affect its composition and structure (Figure 3).

Role of Hepatic Stellate Cells

Chronic liver inflammation and injury are common initiating events in the pathogenesis of liver cirrhosis [34]. Hepatic stellate cells (HSCs), ordinarily dormant in the liver, become active in response to damage and inflammation [35]. After HSCs, the primary factor in the excessive creation of ECM components is activated; they change into cells resembling myofibroblasts and release excessive ECM substances, resulting in fibrosis, which then advances to liver cirrhosis.

Impact on Liver Architecture and Consequences of ECM Accumulation

**Figure 4:**
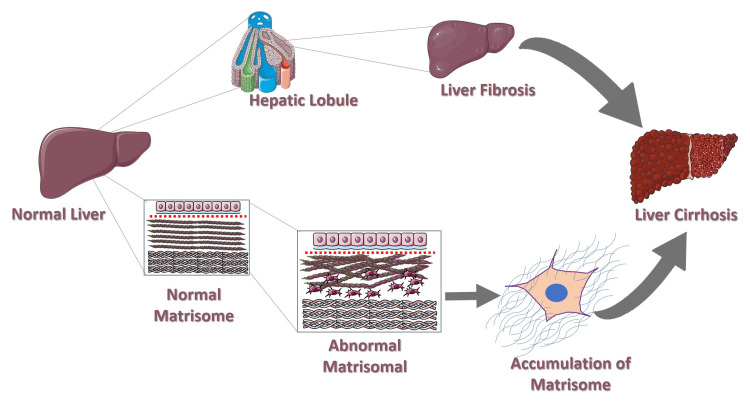
Matrisome disruption, accumulation of extracellular matrix, and fibrosis leading to advanced liver cirrhosis Credit: Adapted from Servier Medical Art [8], licensed under Creative Commons Attribution License (CC BY 4.0).

Increased synthesis and deposition of ECM disrupt the normal liver architecture. Cirrhosis is characterized by persistent fibrosis, which distorts the hepatic vasculature and parenchymal structure [36]. As fibrosis progresses, the liver’s functional capacity becomes increasingly impaired. ECM abnormalities persist, and normal liver functions such as detoxification, protein synthesis, and metabolism are compromised [37]. The continued progression of fibrosis and ECM remodeling contributes to the development of portal hypertension, as increased intrahepatic resistance elevates blood pressure within the portal vein [38]. Alteration of the matrisome plays a central role in the development of these consequences, which include ascites, esophageal varices, and an increased risk of hepatocellular cancer in patients with cirrhosis. Increased fibrogenesis, increased density and stiffness of the liver, and increased resistance to fibrinolysis are all effects of excessive ECM deposition and crosslinking [39]. Therefore, the imbalance between fibrogenesis and fibrolysis can cause liver cirrhosis (Figure 4).

Matrisome alterations in HCC

Matrisome Components in Tumorigenesis

The ECM encompasses the matrisome. The ECM is a dynamic, intricate assemblage of hundreds of proteins that control tissue growth, homeostasis, and cellular metabolism. Numerous studies have established that quantitative and compositional changes in the ECM are closely associated with tumor development [40-45]. Modifications to ECM components have been shown to influence tumor progression and cellular metabolism [46].

Alterations in the matrisome are strongly implicated in the initiation and progression of tumorigenesis [47-49]. Collagen, the most abundant protein in the ECM, exists in multiple subtypes. Increased fibrillar collagen deposition, which directly promotes tumor growth, has been seen in liver tumorigenesis [50,51]. Fibronectin, a major glycoprotein involved in cell adhesion and migration, plays a key role in remodeling the interstitial matrix. Its accumulation contributes to desmoplasia, a fibrotic phenotype that mirrors the pathological changes observed during organ fibrosis. Laminin, an essential component of the basement membrane, is another glycoprotein implicated in cancer progression. In individuals with pancreatic ductal adenocarcinoma, cytoplasmic expression of the laminin two chain is associated with postoperative hepatic metastases and a poor prognosis. Glycosaminoglycan chains and a core protein make up proteoglycans. Proteoglycan levels are higher in liver tumorigenesis [52].

Molecular Mechanism

Several molecular pathways regulate the changes that the matrisome undergoes during tumorigenesis. Integrins, transmembrane receptors that control cell-ECM interactions, are one of the ways. Cell adhesion, migration, and proliferation are all regulated by integrin signaling, which is essential. Different malignancies, including liver cancer, have been linked to altered integrin signaling. Genetic changes in ECM genes can directly affect the structure and functionality of the ECM. For instance, leiomyomatosis has been associated with mutations in collagen genes [53]. A different method is the activation of matrix metalloproteinases (MMPs), an enzyme family that breaks down ECM proteins. MMPs are engaged in several cellular activities, including cell migration, invasion, and ECM remodeling. MMPs play a role in tumor growth and are increased in several malignancies, including liver cancer. ECM is altered due to the hypoxia and acidosis that define the TME. The expression of ECM proteins is changed, and ECM-degrading enzymes are encouraged to be expressed. By producing oxygen-free radicals and secreting cytokines, infiltrating inflammatory cells also contribute to the dynamic changes in the ECM within the TME and ECM remodeling. Different molecular systems that control actions, including cell adhesion, migration, invasion, and angiogenesis, cause matrisome changes during liver tumorigenesis [54-57].

Impact on Tumor Progression

Progression of liver cancer is significantly impacted by changes to the matrisome [58,59]. The ECM also plays a significant role in the metastatic process by encouraging the growth, survival, and invasion of cancer and altering the behavior of fibroblasts and immune cells to promote metastasis [60]. Notably, structural proteins such as collagens I, IV, and V, along with fibronectin and elastin, undergo significant alterations in response to acute toxic liver injury. Tumor cells can invade surrounding tissues and intravasate into capillaries due to changes in the matrisome. The TME is impacted by a disorganized ECM, which makes cancer cells more sensitive to mechanical stress [61]. There are 80 ECM genes (hepatitis B virus (HBV)-related HCC matrisome genes (HHMGs)) that consistently exhibit dysregulation in HBV-related liver cancer, suggesting their participation in disease pathogenesis. These HHMGs are primarily expressed in hepatocytes and endothelial cells, highlighting their function in liver structure and the emergence of cancer [62].

Interplay between matrisome alterations, cirrhosis, and liver cancer

The relationship between cirrhosis, matrisome abnormalities, and liver cancer is complex and remains incompletely understood. However, cirrhosis, which is a risk factor for liver cancer, can occur as a result of alterations in the matrisome. Matrisome modification, which affects cellular proliferation, migration, or invasion, is a characteristic of liver fibrosis and happens during tumorigenesis. 

Transcription factors such as peroxisome proliferator-activated receptors (PPAR)γ, retinoic acid receptors (RARs), retinoid X receptors (RXRs), the pregnane X receptor (PXR), and the LHX2 gene help maintain the quiescent phenotype of HSCs, while others, including Kruppel-like factor 6 (KLF6), Gα-interacting, vesicle-associated protein/Girdin (GIV/Girdin), and methyl-CpG-binding protein 2 (MeCP2), drive the transformation of quiescent HSCs into myofibroblasts. HSCs are kept dormant by normal PPAR function, but decreased PPAR expression in these cells is linked to the development of liver fibrosis and elevated collagen synthesis [63]. There is a connection between the matrisome (genes connected to the ECM) and cancer, particularly liver cancer brought on by the HBV. The research findings are consistent with the following. The study demonstrates a remarkable gene expression imbalance, with a distinct prevalence of down-regulated genes, in HBV-related liver cancer. Hence, these ECM genes contribute to the molecular alterations linked to the onset of cancer [64].

Eighty HHMGs consistently exhibit dysregulation in HBV-related liver cancer, suggesting their participation in disease pathogenesis. These HHMGs are primarily expressed in hepatocytes and endothelial cells, highlighting their function in liver structure and the emergence of cancer. Cirrhosis and liver cancer exhibit a well-documented inverse connection. The matrisome is in charge of preserving an organ's mechanical characteristics and tissue organization. Organ-specific tissue architectures are established by enzymes like lysyl oxidase, which crosslink macromolecules like collagen IV and laminin [65]. The fact that more than 80% of HCCs occur in cirrhotic or fibrotic livers raises the possibility that liver fibrosis plays a significant role in the emergence of hepatic cancer [66].

Role of matrix metalloproteinases

MMPs are zinc-dependent endopeptidases that degrade various ECM proteins. MMPs constitute a diverse family of enzymes with varied physiological functions, including roles in diseases such as cancer metastasis and inflammation [67]. MMPs have a propeptide sequence, a catalytic metalloproteinase domain with catalytic zinc, a hinge region or linker peptide, and a hemopexin domain [68].MMP family members are categorized into subgroups such as gelatinases, stromelysins, collagenases, membrane-type (MT) MMPs, and other MMPs based on substrate preference and protein domain considerations. However, substrate specificity is significantly overlapping between subgroups [69]. MMPs like MMP1 and MMP9 are involved in cancer development by degrading ECM components, promoting tumor invasion, and metastasis [70]. In the process of tumor progression, MMP-2 and MMP-9 can mediate the invasion of tumor cells into the basement membrane through the degradation of collagen IV, thus resulting in tumor metastasis and diffusion [71]. They also promote tumor growth by degrading matrix barriers and enhancing angiogenesis [72]. Some of the mechanisms of MMPs contributing to liver cirrhosis are mentioned here.

ECM Degradation and Fibrosis Progression

MMPs are known for deleting various components of the ECM proteins. In the context of liver cirrhosis, sustained liver injury leads to the activation of HSCs, which are the primary source of ECM-producing myofibroblasts [73]. MMPs, particularly MMP-2, MMP-9, and MMP-13, are upregulated in response to inflammatory and profibrotic signals, promoting ECM degradation and turnover [74,75]. Excessive MMP-mediated ECM degradation disrupts the balance between ECM synthesis and degradation, leading to progressive fibrosis and scar formation in the liver.

Activation of Pro-fibrotic Pathways

MMPs directly degrade ECM components and modulate signaling pathways involved in fibrogenesis. MMPs, such as MMP-2 and MMP-9, cleave latent TGF-β complexes, releasing active TGF-β, a potent pro-fibrotic cytokine [76,77]. Active TGF-β stimulates HSC activation, ECM synthesis, and the production of tissue inhibitors of metalloproteinases (TIMPs), creating a positive feedback loop that sustains fibrogenic responses in the liver [78].

Promotion of Inflammatory Responses

MMPs play a crucial role in orchestrating inflammatory responses within the liver microenvironment, contributing to the pathogenesis of cirrhosis. MMPs, such as MMP-1 and MMP-9, facilitate leukocyte recruitment and infiltration into the liver parenchyma by degrading ECM components and basement membrane proteins [79]. This leads to the release of pro-inflammatory cytokines and chemokines, perpetuating the inflammatory milieu and exacerbating liver injury. Moreover, MMP-mediated proteolysis of cell adhesion molecules and cytokine receptors enhances the activation and responsiveness of inflammatory cells, further amplifying inflammatory signaling pathways [80].

Remodeling of the Pericellular Matrix

In addition to ECM degradation, MMPs remodel the pericellular matrix (PCM), a specialized ECM compartment surrounding cells [81]. PCM remodeling is critical for cellular behavior, including migration, proliferation, and survival. In liver cirrhosis, MMP-mediated alterations in PCM composition and organization modulate HSC activation, hepatocyte apoptosis, and immune cell function. Disruption of PCM integrity by MMPs promotes aberrant cell-cell and cell-ECM interactions, contributing to fibrosis progression and liver dysfunction [82].

Angiogenesis and Vascular Remodeling

MMPs regulate angiogenesis and vascular remodeling processes, which are dysregulated in liver cirrhosis. MMPs degrade ECM components in the vascular basement membrane, facilitating endothelial cell migration and vessel sprouting. Additionally, MMP-mediated proteolysis of angiogenic factors and their receptors modulates endothelial cell function and neovascularization [83].

Role of TIMPs

TIMPs are proteins that inhibit the activity of MMPs, which, in turn, degrade the ECM. TIMPs play a crucial role in maintaining ECM homeostasis and modulating tissue remodeling processes. The liver is particularly sensitive to an imbalance between MMPs and TIMPs in relation to the development of fibrosis and cirrhosis because, for example, overexpression of TIMPs would inhibit ECM degradation, and fibrolysis would result in collagen deposition and scarring. Therefore, understanding the role of TIMPs is essential to elucidate the molecular mechanisms of liver cirrhosis and HCC development.

Inhibition of MMP Activity and ECM Accumulation

TIMPs act as endogenous inhibitors of MMPs, regulating their activity and ECM turnover. By binding to MMPs in a 1:1 stoichiometric ratio, TIMPs prevent excessive ECM degradation and maintain tissue integrity. Dysregulation of TIMPs, characterized by decreased expression or altered activity, is observed in liver pathologies, contributing to ECM remodeling and disease progression. Overproduction of TIMPs, especially TIMP-1 and TIMP-2, reduces ECM degradation.

Hepatic Stellate Cell Activation

TIMPs can influence the behavior of HSCs, key effectors in liver fibrosis. TIMP-1 promotes the survival of activated HSCs by inhibiting apoptosis, thereby sustaining their fibrogenic activity. Additionally, TIMP-1 and TIMP-2 regulate HSC proliferation [84].

Regulation of TGF-β Signaling

TGF-β is a major pro-fibrogenic cytokine that drives HSC activation and ECM production. For instance, TIMP-1 has been shown to interact with TGF-β signaling components, potentially enhancing the fibrogenic response [85].

TME Modulation

TIMP-1 and TIMP-2 can promote the proliferation and survival of cancer cells, such as TIMP-1, which has been shown to activate intracellular signaling pathways such as the phosphatidylinositol 3-kinase (PI3K)/Akt pathway, which promotes cell survival and proliferation [86,87].

Interaction With Growth Factors

TIMPs can interact with and modulate the activity of various growth factors involved in tumorigenesis. TIMP-1, for example, can bind to CD63/integrin complexes on the cell surface, leading to the activation of cell survival pathways [88].

Influence on Cell Migration and Invasion

By inhibiting MMP activity, TIMPs can reduce the degradation of the ECM, thereby limiting tumor cell invasion and metastasis. However, the overexpression of TIMPs can paradoxically promote tumor progression by creating a more rigid and supportive TME [89].

Role of immunotherapy in HCC and matrisome alterations

HCC therapy’s latest addition is immunotherapy, which relies on the changes in the TME due to matrisome changes. Nivolumab and pembrolizumab are the immune checkpoint inhibitors (ICIs) with the most significant focus in research for advanced HCC. They work by activating T lymphocytes so that they can target and destroy malignant cells. Immune response and cell invasion are modulated by the β-glucocerebrosidase enzyme (βGC) matrisome component, the ECM. Tumor-associated collagen and fibronectin, as ECM proteins, serve as physical blockades to immune cell infiltration into the tumor. Moreover, during ECM remodeling, bioactive factors that suppress immune responses are also produced. These obstacles can be bypassed through ICI and ECM-targeted therapies, enabling such approaches to complement immunotherapy in the effective management of HCC [90,91].

Gene therapy and RORγ receptor-targeting therapy

Modern approaches can be employed to tackle the underlying molecular pathways in HCC due to advancements in gene therapy. The retinoic acid-RORγ receptor, a nuclear receptor that modulates immune responses and plays a role in tumorigenesis, has been pinpointed as a therapeutic target. The objective of RORγ receptor therapy is to alter immune pathways to improve the anti-tumor immune response and block pro-tumorigenic pathways. Recent publications indicate that RORγ targeting may have an impact on ECM remodeling and traits of the tumor microenvironment, leading to diminished tumor growth and metastasis. Creating gene therapies dedicated to this receptor may ultimately result in new management strategies for liver cancer [92].

Emerging therapeutic strategies targeting matrisome alterations in liver cirrhosis and cancer

Two hundred seventy-eight genes, or 1% of the human proteome [93], were discovered through proteomic investigations of the in vivo ECM composition and in silico prediction as being essential components of the "matrisome" for humans. In the presence of cirrhosis and other risk factors for liver cancer, the matrisome, a crucial component of the hepatic microenvironment, may change. ECM targeting promising therapeutic targets for cancer therapy includes the ECM. Targeting the ECM may be a viable therapeutic approach for liver cancer, as changes in the ECM can contribute to the development of the disease [93].

Anti-fibrotic Tactics

Hepatic matrisomes undergo qualitative and quantitative changes during fibrosis, and focusing on these modifications could be a possible anti-fibrotic tactic. Research is currently focused on new treatments and strategies that target matrisome changes in cancer and liver cirrhosis. Promising therapeutic targets for cancer therapy include the ECM. Targeting the ECM may be a viable therapeutic approach for liver cancer, as changes in the ECM might contribute to the development of the disease. Using immunotherapy to treat HCC has shown promise. In clinical studies for advanced HCC, ICIs like nivolumab and pembrolizumab have been studied. These medications try to use the immune system to combat cancer cells.

Matrisome Marker Identification

Statistically reliable matrisome markers for tumor subtypes have been found using machine learning algorithms; these markers can be used to create novel, tailored therapy strategies. Future treatments that target interactions between cells, soluble mediators, the ECM and its receptors, and pertinent intracellular signals show great promise for treating liver fibrosis in a multifaceted manner [94].

Current research and future directions

In matrisome research, ECM data from 17 studies on the ECM of 15 distinct tissues are available in the searchable database matrisomeDB [95]. Fibrotic tumors in ovarian cancer have been studied using matrisome expression signatures [96]. Finding ECM proteins connected to critical developmental or pathological processes is one of the future research topics. To activate the immune system's defenses against tumor cells, a tissue-engineered matrisome has been employed as an antigenic depot [97]. Comprehensive studies are needed to identify reliable biomarkers, develop targeted therapeutic strategies, and advance our understanding of the matrisome’s involvement across a spectrum of pathological conditions.

## Conclusions

Our comprehensive review of the literature suggests that alterations in the matrisome play a pivotal role in both tumorigenesis and liver cirrhosis. Studies have demonstrated that the hepatic matrisome responds dynamically to injury, with the qualitative and quantitative changes observed during fibrosis extending far beyond the mere accumulation of collagen. Proteomic analyses of matrisome composition have identified distinct matrisomal proteins that correlate with early cirrhosis progression and patient survival. This review highlights that a deeper understanding of the hepatic matrisome and its response to injury may provide novel mechanistic insights into both disease progression and regression. Matrisome alterations are driven by multiple interrelated mechanisms, including immune responses, genetic mutations, and aberrant matrisomal organization. Due to the complexity and interconnected nature of these pathways, further investigation is essential to fully elucidate their roles in cancer development. The matrisome encompasses genes encoding ECM components that provide structural support to tissues such as the liver. Chronic liver injury, including cirrhosis, induces structural and compositional changes in the ECM through matrisome dysregulation.
